# Leveraging the power of pooled data for cancer outcomes research

**DOI:** 10.1186/s40880-016-0132-0

**Published:** 2016-08-02

**Authors:** Kiara Hugh-Yeun, Winson Y. Cheung

**Affiliations:** Division of Medical Oncology, British Columbia Cancer Agency, 600 West 10th Avenue, Vancouver, BC V5Z 4E6 Canada

**Keywords:** Colon cancer, Informed consent, Risk assessment, Clinical outcomes

## Abstract

**Background:**

Clinical trials continue to be the gold standard for determining the efficacy of novel cancer treatments, but they may also expose participants to the potential risks of unpredictable or severe toxicities. The development of validated tools that better inform patients of the benefits and risks associated with clinical trial participation can facilitate the informed consent process. The design and validation of such instruments are strengthened when we leverage the power of pooled data analysis for cancer outcomes research.

**Main body:**

In a recent study published in the *Journal of Clinical Oncology* entitled “Determinants of early mortality among 37,568 patients with colon cancer who participated in 25 clinical trials from the adjuvant colon cancer endpoints database,” using a large pooled analysis of over 30,000 study participants who were enrolled in clinical trials of adjuvant therapy for early-stage colon cancer, we developed and validated a nomogram depicting the predictors of early cancer mortality. This database of pooled individual-level data allowed for a comprehensive analysis of poor prognostic factors associated with early death; furthermore, it enabled the creation of a nomogram that was able to reliably capture and quantify the benefit-to-risk profile for patients who are considering clinical trial participation. This tool can facilitate treatment decision-making discussions.

**Conclusion:**

As China and other Asian countries continue to conduct oncology clinical trials, efforts to collate patient-level information from these studies into a large data repository should be strongly considered since pooled data can increase future capacity for cancer outcomes research, which, in turn, can enhance patient-physician discussions and optimize clinical care.

## Background

The development of novel cancer diagnostics and therapeutics is largely dependent on valuable insights gained from randomized controlled clinical trials. Although clinical trials remain the gold standard for determining the overall efficacy, feasibility, and safety of these new interventions, they may also expose patients to unnecessary harms since new treatments inherently carry the risk of unpredictable or severe adverse events. Therefore, the benefits and risks of clinical trials must be carefully balanced, and patients who are considering study participation should engage in thorough discussions with their physicians before providing informed consent. Similarly, clinicians must weigh the pros and cons of clinical trials to ensure that study participation does not pose excessive harms to patients.

To date, determining whether a patient is suitable for clinical trials has been primarily dependent on a combination of patient preference and clinician judgement. However, this approach can be potentially unreliable, especially for specific subpopulations that are either older or frailer. The development of readily accessible and user-friendly tools that objectively inform patients of the benefits and risks associated with clinical trial participation can facilitate the informed consent process as well as the patient-physician conversation. The design and validation of such instruments are strengthened when we leverage the power of pooled data analysis for cancer outcomes research.

The use of large databases of pooled patient data has already made it possible to address several important and clinically relevant research questions. Data from real-world, population-based settings [e.g., the British Columbia Cancer Agency (BCCA)] and clinical trial settings [e.g., Adjuvant Colon Cancer Endpoints (ACCENT)] have been previously interrogated to generate high-profile articles that successfully examined the effect of systemic therapy on overall and cancer-specific survival as well as the effect of various clinical and pathologic factors, such as age, race, and stage, on outcomes [1–3].

Whereas previous studies have largely explored prognostic factors for early mortality among phase I study participants, similar studies to identify prognostic factors among phase III study participants have not been conducted. Most prior research was further limited by small sample sizes or significant heterogeneity in the pooled cohorts [4, 5]. Thus, we saw an opportunity to use the ACCENT database to identify prognostic factors related to early mortality in phase III study participants. Specifically, the ACCENT database represents a large pooled repository of individual-level data from patients who had previously participated in phase III adjuvant colon cancer clinical trials. Leveraging this comprehensive database, we were able to provide insights into prognostic factors for early mortality in phase III study participants.

## Main body

In our recent *Journal of Clinical Oncology* article entitled “Determinants of early mortality among 37,568 patients with colon cancer who participated in 25 clinical trials from the adjuvant colon cancer endpoints database” [6], we reported the results of a study that characterized the determinants of early mortality in a large cohort of early-stage colon cancer patients who had participated in prior adjuvant clinical trials. This study was conducted because the factors associated with early death after surgery and adjuvant chemotherapy are poorly defined. Therefore, we conducted a pooled analysis of over 30,000 patients from 25 randomized clinical trials of adjuvant systemic therapy. Using multivariate logistic regression models and controlling for confounders, we successfully developed and validated a nomogram for 6-month mortality.

We found that early mortality was very low: 0.3% at 30 days, 0.6% at 60 days, 0.8% at 90 days, and 1.4% at 6 months [6]. Consistent with other studies [7–9], our prognostic analyses showed that advanced age, male gender, worse performance status, and higher tumor stage and grade predicted a greater likelihood of early mortality, whereas treatment received was not significantly associated with early mortality. Our findings underscored the observation that early mortality was generally uncommon, but it was more frequently seen in specific subsets of patients, such as those who were older and frailer. This highlights the importance of tools that can better clarify the benefit-to-risk ratio to patients who are considering clinical trial participation. The nomogram developed from our analysis has been validated and calibrated to serve as a potentially effective instrument that can guide and enhance treatment decision-making and discussions between clinicians and patients.

This study is also a proof-of-principle for other countries in terms of illustrating the strength of large databases. Colon cancer is a common cancer worldwide [10]. Particularly in China, the incidence of colon cancer is anticipated to increase significantly over time, and the burden of this disease and its effect on society are expected to grow exponentially, especially given the longer lifespan of patients that has resulted from recent diagnostic and therapeutic advances [11, 12]. Although clinical trials are offered globally, the number of phase III trials available in China often pales in comparison to the number available in Western countries. Reasons for this disparity are numerous and may include various clinical and systemic factors, such as infrastructure and resource constraints or concerns regarding the risks of adverse events that may be more prevalent or unique among Asian patients.

As in many other countries, in China participation in clinical trials is suboptimal. It is estimated that only 2%–4% of patients in China with cancer ultimately consent to enroll in clinical trials, even when studies are available and offered to eligible patients [13]. This finding has been attributed in part to inherent cultural beliefs that regard clinical trials as socially undesirable [14]. Interestingly, these negative perceptions of clinical trials have been shown to dissipate after effective educational interventions and open discussions with physicians [15]. As such, our nomogram may aid clinicians in their conversations with patients.

Because large clinical trials are not always accessible, and since important research questions almost always require adequate sample sizes to address well, pooling data from either population-based settings or clinical trial settings is an effective strategy for cancer outcomes research. Currently, the ACCENT database comprises patient-level data from over 25 adjuvant colon cancer clinical trials from North America and Europe. When the outcome of interest, such as early mortality, is relatively rare, pooling data can add validity to the analysis and strength to its findings. Presently, China is not part of similar database-driven initiatives, but pooling of such data may prove to be an invaluable resource for Asian patients [16]. Databases such as those available at BCCA or via ACCENT are excellent examples of the power of pooled data. The creation of similar databases of Asian patients should be strongly encouraged to make investigations of rare but clinically pertinent endpoints more feasible [17].

## Conclusions

We developed and validated a ready-to-use and user-friendly nomogram to assist potential clinical trial participants with benefit-to-risk assessments. The ability to quantitatively and objectively predict the risk of harm and early death can better facilitate the patient-physician dialogue and the informed consent process. For this study, we used a large database of pooled individual-level data from patients who previously participated in clinical trials [6]. Having access to such a database of pooled information will continue to be invaluable for investigators who address other important research questions. As China and other Asian countries continue to conduct oncology clinical trials, efforts to collate patient-level information from these studies into a large data repository should be strongly considered since pooled data can increase future capacity for cancer outcomes research, which, in turn, can enhance patient-physician discussions and optimize clinical care. More importantly, the development of a database initiative specific to Chinese patients will further enable researchers to study aspects of cancer care that may be unique to Asia.
